# Microstructure and Corrosion Resistance of PEO Coatings Formed on KBM10 Mg Alloy Pretreated with Nd(NO_3_)_3_

**DOI:** 10.3390/ma11071062

**Published:** 2018-06-22

**Authors:** Junpeng Lu, Xing He, Hongxia Li, Renguo Song

**Affiliations:** 1 Jiangsu Key Laboratory of Materials Surface Science and Technology, Changzhou University, Changzhou 213164, China; jpgege163@163.com (J.L.); hx_ccu@163.com (X.H.); 2 Jiangsu Collaborative Innovation Center of Photovolatic Science and Engineering, Changzhou University, Changzhou 213164, China; 3 College of Materials and Environmental Engineering, Hangzhou Dianzi University, Hangzhou 310018, China; hxli@hdu.edu.cn

**Keywords:** plasma electrolytic oxidation, pretreatment, microstructure, corrosion resistance, magnesium alloy, Nd(NO_3_)_3_ solution

## Abstract

Plasma electrolytic oxidation (PEO) technique is one of the important methods used in the surface modification of magnesium alloys. In this paper, the ceramic coatings on pretreated KBM10 magnesium alloy with Nd(NO_3_)_3_ solution were prepared by PEO. The effects of Nd(NO_3_)_3_ solution concentration on the microstructure and corrosion resistance of PEO coatings on magnesium alloys were investigated by means of scanning electron microscopy (SEM), X-ray diffractometer (XRD), and electrochemical workstation. It was found that the surface of the coatings was porous after PEO, and element Nd could be deposited on the surface of the coatings by pretreatment and existed in the PEO coatings. The coating formed at Nd(NO_3_)_3_ solution concentration of 0.06 mol/L exhibited the best corrosion resistance among all the as-prepared coatings.

## 1. Introduction

Magnesium alloys have great application value in the field of materials. Because of urgent energy and environmental problems [1], and the obvious advantages [2] of magnesium alloys, magnesium alloys have been applied in many fields: the automobile industry, the 3C industry, the aerospace industry, metallurgy, the chemical industry, modern weapons, the nuclear industry, and so on [3,4,5]. The application range of magnesium alloys has been continuously expanding, but the high chemical activity and mechanical strength of magnesium have constrained the development of magnesium alloys [6,7]. The standard equilibrium potential of Mg/Mg^2+^ is −2.34 V [8], so it is highly susceptible to chemical and electrochemical corrosion. At the same time, the corrosion potential of magnesium alloy in aqueous solution is also negative, about −1.5 V, which is more prone to corrosion than other metallic materials. The corrosion equations of magnesium alloys in water and air are as follows:

(1) $$\mathrm{Mg}+2H_{2}O\to \mathrm{Mg}{\left(\mathrm{OH}\right)}_{2}+H_{2}↑$$

(2) $$\mathrm{Mg}+2H_{2}O+{\mathrm{CO}}_{2}\to {\mathrm{Mg}}_{2}{\mathrm{CO}}_{3}+H_{2}↑$$

The rapid degradation rate of magnesium affects its integrity of magnesium in the environment, and the long-term corrosion protection ability is an important index with which to evaluate the PEO film. Long time immersion test is an intuitive method to evaluate the degradation process and corrosion resistance of the PEO film [9].

Plasma electrolytic oxidation (PEO), also called micro-arc oxidation (MAO), a new surface treatment technology [10,11,12], is attracting more and more attention. PEO technology is mainly used on aluminum [13], magnesium [14,15], titanium [16,17], and other metals [18]. Due to its non-pollution and cleaning characteristics, more and more attention has been paid to the plasma electrolytic oxidation technology. However, there are still a lot of research studies on the corrosion resistance of plasma electrolytic oxidation technology [19,20].

At present, there little research has been carried out on the PEO coatings of rare earth [21]. The addition of rare earth elements to the PEO coatings can improve the microstructure of the film and optimize the corrosion resistance of the film [22]. Moreover, the corrosion resistance of magnesium alloy coating can be improved by PEO after immersion with rare earth salt solution [23]. The authors of [24] show that electrolytes containing rare earth compounds can improve coating thickness, structure, and corrosion resistance. Adding CeO_2_ particles to the oxidation solution can dope rare earth oxide into the PEO film, which can improve the structure of the film and increase the density of the film [25]. In addition, the alloy was pretreated with shot peening and soaking, and then PEO was carried out. The results showed that the prefabricated PEO coating with ultrasonic shot peening rare earth was smoother, and the corrosion resistance of the coating was greatly improved [26]. Cai [27] investigated the microstructure of PEO coatings on prefabricated Ce coatings of magnesium alloys. The results showed that the prefabricated coatings had the ability to improve the smoothness of PEO coatings. An immersion test showed that the PEO ceramic coatings were more corrosion-resistant after prefabricated coating.

The aim of the present work was to study the microstructure and corrosion resistance of PEO coatings on KBM10 magnesium alloy after pretreatment with Nd(NO_3_)_3_ solution in detail.

## 2. Experimental

### 2.1. Materials and Coatings Preparation

In the experiment, the PEO was performed on a rectangular plate of 25 mm × 30 mm × 3 mm. The composition of KBM10 magnesium alloys is shown in Table 1. Before PEO treatment, the specimen was polished with SiC abrasive paper to a grit of 1000 before anodizing, then washed with ethanol, deionized water, and dried.

Firstly, the Nd(NO_3_)_3_ was added directly to the electrolyte; then, the samples were treated by PEO. However, the coating was not formed on the magnesium alloy surface as expected, and the experiment failed. This result was probably due to Nd(NO_3_)_3_ precipitating in the alkaline electrolyte, and the fact that the Nd element in the precipitated form cannot participate in the PEO process. Therefore, different concentrations of Nd(NO_3_)_3_ solution pretreatment were used instead. The sample was soaked in Nd(NO_3_)_3_ solution for 20 min, and then plasma electrolytic oxidation was carried out.

The preparation method of the coatings was as follows: pretreated solution: Nd(NO_3_)_3_ mol/L; electrolyte: Na_2_SiO_3_ 10 g/L, KOH 5 g/L, Al_2_O_3_ 2 g/L, and NaF 0.5 g/L. PEO device setting (JHMAO-20H, Xi’an Jin Tang Material Application Technology Co., Ltd., Xi’an, China): constant voltage mode: 350 V; duty cycle: 4 min; work frequency: 400 Hz; and treatment time: 15 min. The substrates were used as anode, and stainless steel plate was treated as cathode. The prepared samples were cleaned with deionized water and dried in ambient air.

### 2.2. Coatings Characterization

The thickness of the coatings was measured by the TT240 thickness meter (Beijing Time Technologies Co., Ltd., Beijing, China). Take 5 points on the surface and calculate the average value.

A scanning electron microscope (SEM) (JSM-6510, JEOL Ltd., Tokyo, Japan) was used to characterize the surface and cross-section morphology of the PEO coatings. Some specimens were cross-sectioned and subsequently mounted in resin and polished using a standard metallographic abrasive paper for the observation of cross-section morphology of the coatings. Energy dispersive X-ray spectrometer (EDS, JEOL Ltd., Tokyo, Japan) was employed to analyze the element in the PEO coatings.

The phase compositions of the PEO coatings were determined using X-ray diffraction (XRD, Rigaku Corporation, Tokyo, Japan) with Cu Ka radiation between 2θ values of 20° and 90° with a step length of 0.02 at a scanning rate of 1/min. The X-ray generator setting was 40 kV and 100 mA.

The corrosion resistance of samples before and after plasma electrolytic oxidation was mainly evaluated by potentiodynamic polarization curve and EIS test. The samples were immersed in 3.5 wt % NaCl (pH = 7) solution at room temperature of 298.2 ± 1 K with an exposed area of 1 cm^2^. The potentiodynamic polarization was measured by CS350 electrochemical workstation (Wuhan Corrtest Instrument Co. Ltd., Wuhan, China). The electrochemical working system equipped with a standard three-electrode cell, saturated calomel electrode as reference electrode, and a standard platinum electrode as auxiliary electrode (also called opposite electrode); the sample was working electrode, and the test temperature was 25 ± 1 °C. The sample was immersed in 3.5 wt % NaCl solution for 0.5 h. Electrochemical impedance spectroscopy (EIS) was performed over the frequency range of 10^−2^–10^5^ Hz with an AC signal amplitude of 10 mV with respect to OPC. The potentiodynamic polarization should be immersed in 3.5 wt % NaCl solution for about 30 min until the open circuit potential was stable. The test should be repeated five times each time to ensure the repeatability and reliability of the test results.

### 2.3. Degradation Behavior Examination

The samples were immersed in 3.5 wt % NaCl solution at room temperature in order to further evaluate the corrosion resistance and degradation performance of the PEO coatings. The ratio of corrosion area to immersion solution’s volume was 1 cm^2^/50 mL. The corrosion solution was changed every 24 h. After soaking, the surface and cross section of the coating were sprayed with Pt and investigated by means of scanning electron microscopy. The degradation of the coatings was judged according to the surface and section micro-morphology of the coatings. At the same time, the coatings were tested by XRD after immersion, and the chemical composition of the coatings were analyzed. The corrosion resistance of the coatings was evaluated by impedance spectroscopy. The test should be repeated five times to ensure the repeatability and reliability of the test results. Finally, a coating degradation model was established to analyze the degradation process.

## 3. Results and Discussion

### 3.1. Morphology and Composition

**Figure 1:**
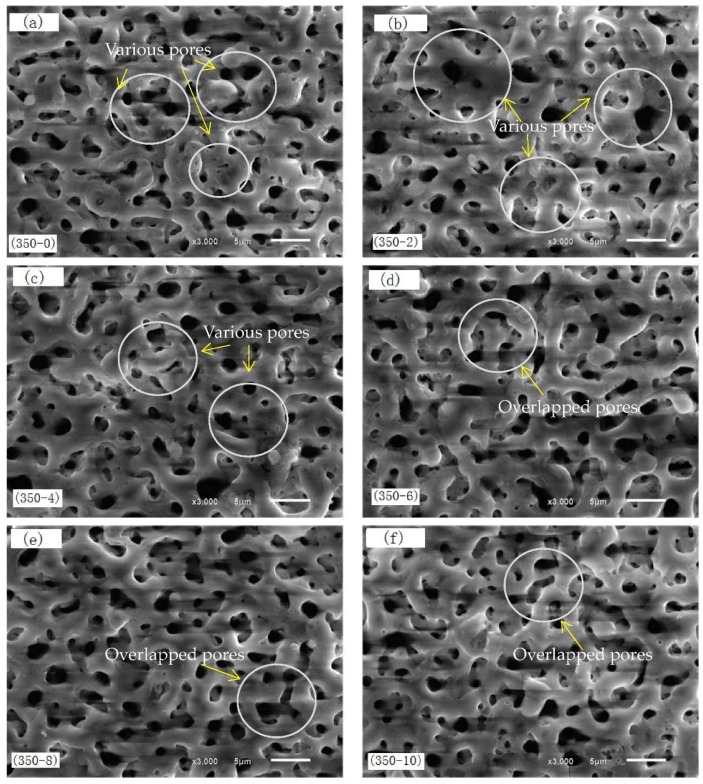
Surface morphology of micro-arc oxidation (MAO) coatings formed at pretreatment with different concentrations of Nd(NO_3_)_3_ solution: (**a**) 350–0, (**b**) 350–2, (**c**) 350–4, (**d**) 350–6, (**e**) 350–8, and (**f**) 350–10.

Figure 1 shows the surface morphology of PEO coatings formed at pretreated with different concentrations of Nd(NO_3_)_3_ solution. After PEO treatment, the surfaces of the coatings were porous and evenly distributed. These pores were due to the ejection of gases and melts from the discharge channels. The size of the pore was about 1–3 μm, which is more uniform than that in literature [28]. Due to the addition of the compound NaF in the electrolyte, F^−^ is of ability to increase the uniformity of coatings in PEO treatment [29]. With the increase of the concentration of pretreated Nd(NO_3_)_3_ solution, the surface pores were gradually uniform; individual pores gradually closed, and some pores overlapped (the circle Figure 1d–f), but the change was not obvious. This could be due to the limited degree of oxidation of the substrate using the concentration of Nd(NO_3_)_3_ solution.

**Figure 2:**
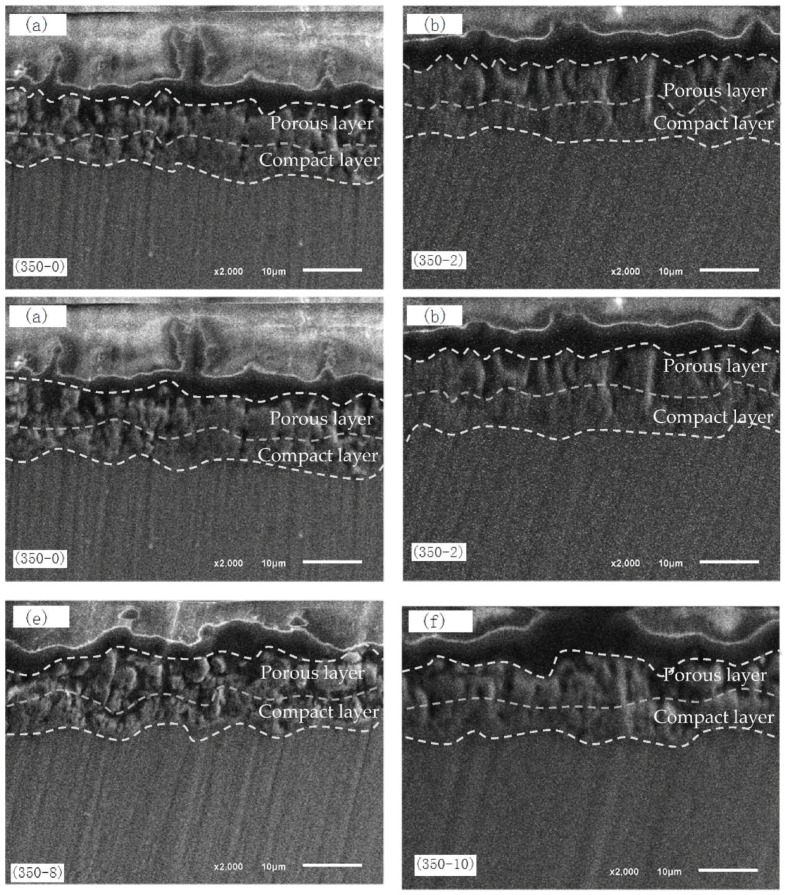
Cross-section morphology of MAO coatings formed at pretreatment with different concentrations of Nd(NO_3_)_3_ solution: (**a**) 350–0, (**b**) 350–2, (**c**) 350–4, (**d**) 350–6, (**e**) 350–8, and (**f**) 350–10.

**Figure 3:**
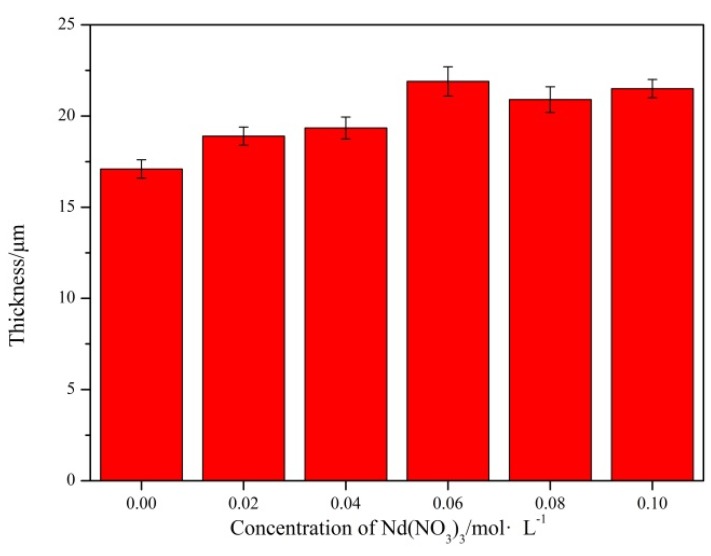
The thickness of MAO coatings.

Figure 2 shows the cross-section morphology of MAO coatings formed at pretreatment of different concentration of Nd(NO_3_)_3_ solution. Figure 3 shows the thickness of MAO coatings. In Figure 2, the coatings bind well to the metal substrate, and the boundary was not obvious. Close to the magnesium alloy substrate, the MAO coatings consisted of an outer porous layer and inner compact layer. Because of the continuous melting and solidification cycle process, there were some holes and cracks in the MAO coatings, which were the discharge channels of the micro-arc oxidation of magnesium alloy; additionally, channels for molten oxide overflow the surface of the coatings.

The (350–0) film layer was thin, and the pore was not uniform. With the increase of the pretreatment concentration of Nd(NO_3_)_3_ solution, the thickness of the film increased gradually during the PEO treatment. This may be the formation of a thin oxide layer on the surface of magnesium alloy after immersion in Nd(NO_3_)_3_ solution, which ensures the surface discharge of Mg alloy is uniform. Figure 2 (350–6) shows the thickest coating; there were few pores in the film layer, and the conductivity of the film layer was weak. 

Figure 3 shows that the cross-section thickness exhibits an increase with the increase in the concentration of Nd(NO_3_)_3_ solution, and the concentration of Nd(NO_3_)_3_ increased to a certain value; the thickness does not increase any more. It can be observed that pretreatment with Nd(NO_3_)_3_ solution can increase the thickness of PEO coatings at a particular range. The optimal concentration for improving the film structure was 0.06 mol/L.

**Figure 4:**
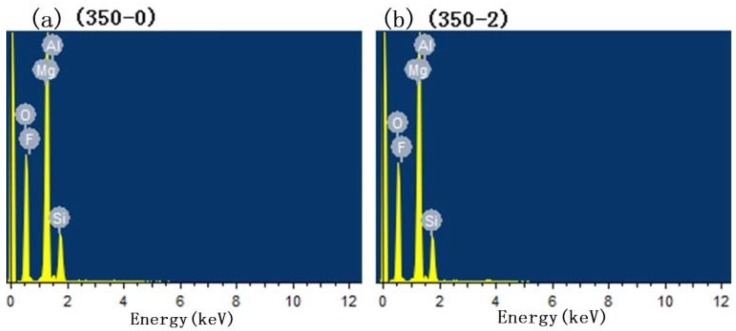

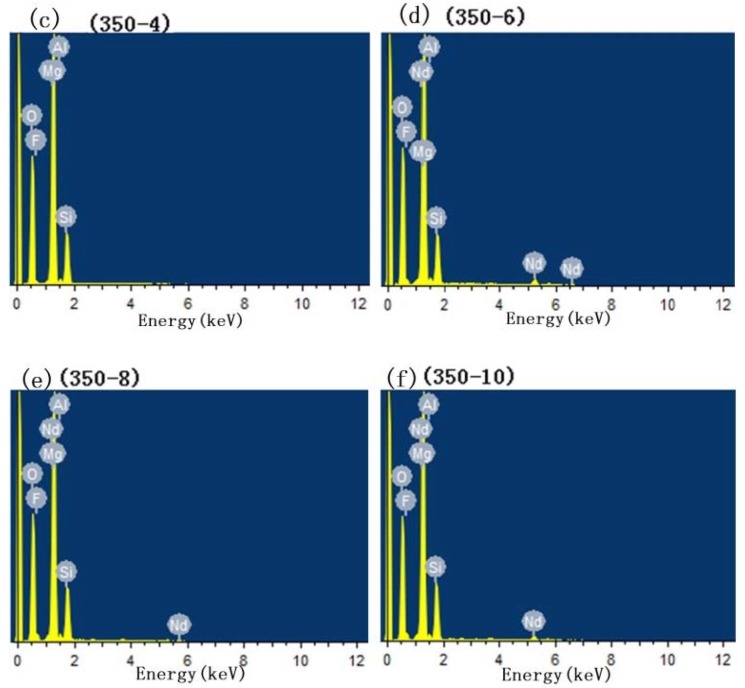
The test result of Energy dispersive X-ray spectrometer (EDS): (**a**) 350–0, (**b**) 350–2, (**c**) 350–4, (**d**) 350–6, (**e**) 350–8, and (**f**) 350–10.

Figure 4 shows the test result of EDS, and the scanning area is in Figure 1. The element content results are shown in Table 2. The results of EDS showed that the main elements of the coatings were Mg, O, F, Si, Al, and Nd. The content of Mg in the coatings gradually decreased with the increase in the concentration of pretreatment Nd(NO_3_)_3_ solution. The content of F and Si increased gradually. This showed that pretreatment with Nd(NO_3_)_3_ solution could obviously increase the reaction between the electrolyte and magnesium alloy, and make the elements in the electrolyte enter into the coatings. When the concentration of Nd(NO_3_)_3_ solution was less than 0.06 mol/L, the coatings contained almost no Nd element, which may be due to the low content of Nd element in the film, which cannot be detected. The (350–6) owned the highest content of Nd, when the concentration was higher than 0.06 mol/L. After the PEO process, the content of Nd in the coatings did not increase with the pretreatment concentration of Nd(NO_3_)_3_, which may be due to the intense reaction that made Nd elements attached to the magnesium alloy surface fall into the electrolyte, which led to the decrease of the content. 

**Figure 5:**
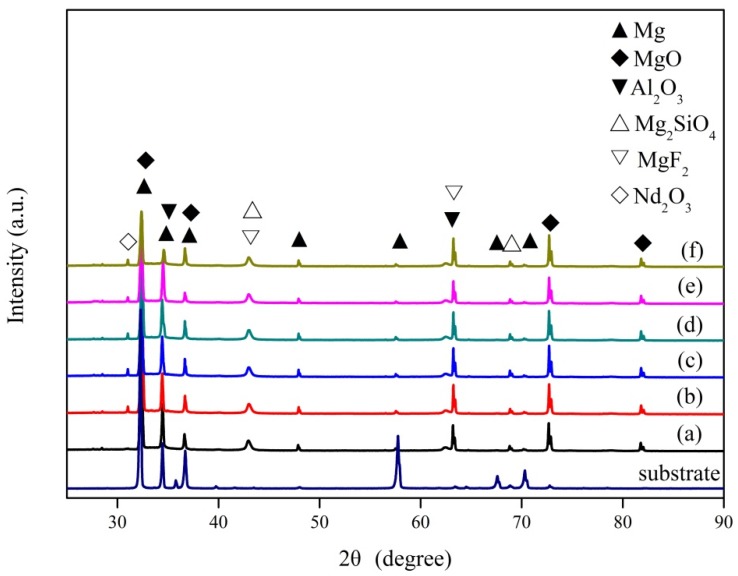
The X-ray diffraction (XRD) patterns of magnesium alloy substrate and MAO coatings at pretreatment of different concentrations of Nd(NO_3_)_3_: (a) 350–0, (b) 350–2, (c) 350–4, (d) 350–6, (e) 350–8, and (f) 350–10.

Figure 5 shows the XRD patterns of magnesium alloy substrate and PEO coatings at pretreatment of different concentrations of Nd(NO_3_)_3_. It is evident from Figure 5a–f that the coatings were mainly composed of MgO, Al_2_O_3_, Mg_2_SiO_4_, MgF_2_, and Nd_2_O_3_. The presence of Mg peak corresponding to the Mg alloy substrate could be due to the penetration of X-ray into the substance owing to the thin coatings. This result showed that the Nd element can enter the coatings by pre-treatment and existed in the film as another Nd compound. This coincided with the presence of Nd element in Table 2. The existence of Mg_2_SiO_4_ indicated that the main salt component in the electrolyte was involved in the formation of the coatings. The appearance of diffraction peaks corresponding to MgF_2_ indicated that NaF in the solution participates in the process of plasma electrolytic oxidation, and the presence of Al_2_O_3_ in the film in this form indicated that Al_2_O_3_ can be deposited into the coatings by means of PEO.

### 3.2. Polarization Test

**Figure 6:**
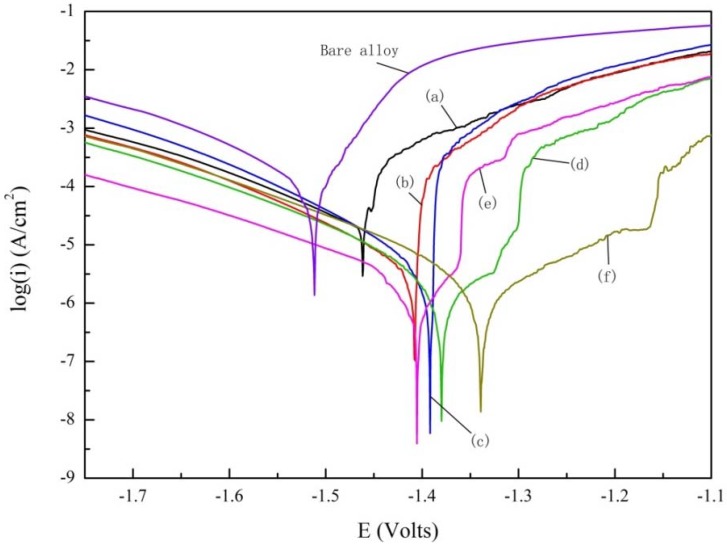
The typical potentiodynamic polarization curves obtained for the substrate and MAO coatings at pretreatment of different concentration of Nd(NO_3_)_3_ in 3.5 wt % NaCl solution: (a) 350–0, (b) 350–2, (c) 350–4, (d) 350–6, (e) 350–8, and (f) 350–10.

In order to evaluate the corrosion resistance of magnesium alloys treated with different concentrations of Nd(NO_3_)_3_ solution, potentiodynamic polarization curves of the coatings and the substrates were measured in 3.5 wt % NaCl solution. The typical potentiodynamic polarization curves obtained for the substrate and PEO coatings at pretreatment of different concentrations of Nd(NO_3_)_3_ in 3.5 wt % NaCl solution are shown in Figure 6. Table 3 represents the electrochemical parameters of the sample in Figure 6. It can be found from the figure that the corrosion potential (*E_coor_*) of the samples prepared by the PEO coatings were all positive shift. The corrosion current density (*I_coor_*) of the sample prepared by PEO coatings in the table was lower than that of the substrate (decrease by more than one order of magnitude), which indicated that the corrosion resistance of the sample treated by plasma electrolytic oxidation is improved significantly [30]. With the increase in the pretreatment concentration of Nd(NO_3_)_3_ solution, the *I_coor_* of the sample decreased to 8.02 × 10−7 μA/cm^2^, then increased slightly, and the polarization resistance (*R_p_*) gradually increased to 1.89 × 107 Ωcm2, then decreased slightly, which is similar to the result in some articles [31,32]. In Table 3, the maximum *I_coor_* decreased by three orders of magnitude relative to the substrate, and the *R_p_* increases by two orders of magnitude relative to the substrate. Compared with (350–6), the corrosion resistance of (350–8) and (350–10) decreased only a little, which can be regarded as the same corrosion resistance. The above polarization tests showed that the PEO coatings pretreated with 0.06 mol/L Nd(NO_3_)_3_ exhibited the best corrosion resistance.

### 3.3. Long Time Immersion Tests

#### 3.3.1. Morphology and Composition

**Figure 7:**
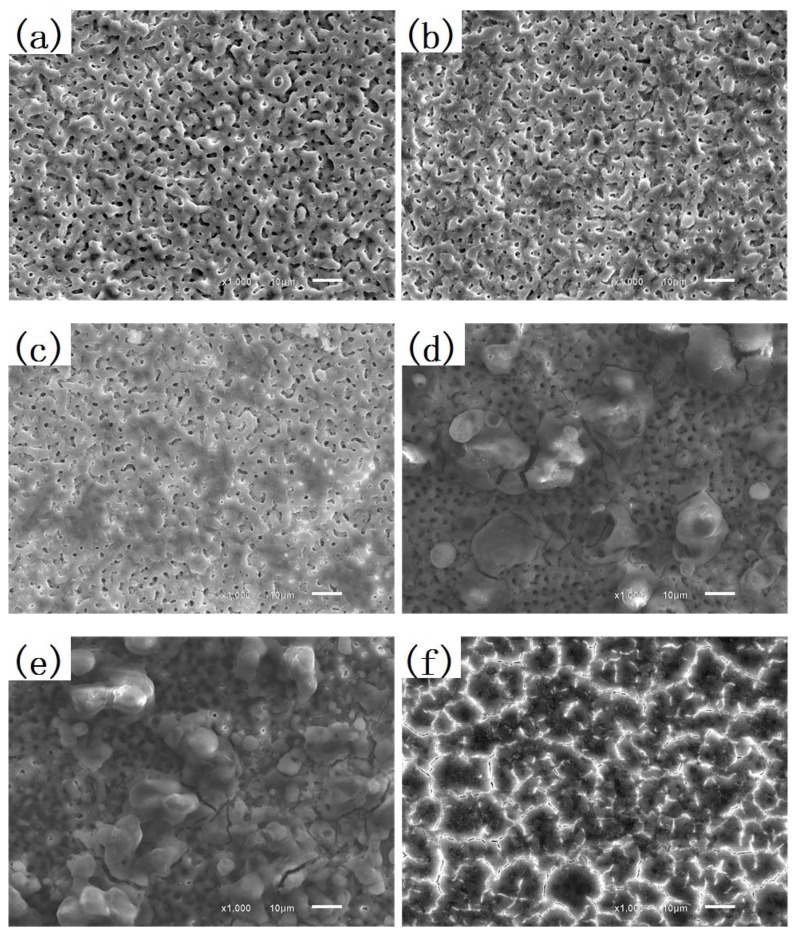
The surface morphology of MAO coatings after immersion in 3.5 wt % NaCl solution: (**a**) 24 h, (**b**) 48 h, (**c**) 96 h, (**d**) 192 h, (**e**) 384 h, and (**f**) 768 h.

**Figure 8:**
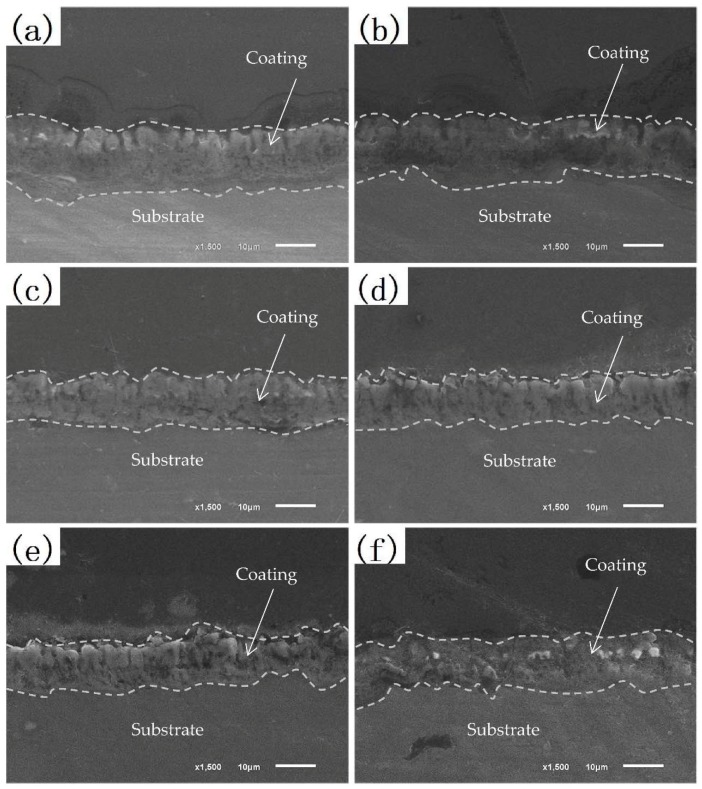
The cross-section morphology of MAO coatings after immersion in 3.5 wt % NaCl solution: (**a**) 24 h, (**b**) 48 h, (**c**) 96 h, (**d**) 192 h, (**e**) 384 h, and (**f**) 768 h.

The cross-section and surface morphologies of PEO coatings after immersion in 3.5 wt % NaCl solution are illustrated in Figure 7 and Figure 8. It was found in Figure 7 that there was little evidence of corrosion on the surface of the coatings after immersion for 96 h. In Figure 7c, the NaCl solution infiltrated into the film through the outer porous layer channel, and the corrosion products accumulated in the pores of the coating so that the pores on the film surface were blocked. This means that the PEO coatings were provided with a good effect with regard to corrosion prevention. After immersion for 24 h (Figure 8a), the thickness of the coating was almost the same as that of the unsoaked sample. The thickness of the coatings began to decrease, and the pores on the surface of the coatings expanded after immersion for 48 h. This demonstrates that there was slight corrosion on the surface at the initial stage of soaking; the loose layer surface was corroded off, which reduced the thickness of the coating. However, the thickness of the coatings did not decrease further after immersion for 96 h, which showed the increase of surface corrosion particles, so that the corrosion products deposited on the surface of the coatings, and the thickness of the coatings was no longer reduced.

After the immersion time increased to 384 h, the pore channel on the surface of the film enlarged, more and more corrosion products were deposited, and cracks appeared on the surface of the coating. From Figure 8e, it can be seen that the pore channels of the coating gradually extended to the substrate, and the appearance of the cracks (Figure 7e) provided a channel for the corrosion solution to enter the substrate surface. It can be inferred that the corrosion solution has penetrated into the coating and caused the partial rupture of the coating. With the soaking time reaching 768 h (Figure 7f and Figure 8f), the coating thickness decreased to about 10 μm, the numerous surface cracks indicating signs of exfoliation.

**Figure 9:**
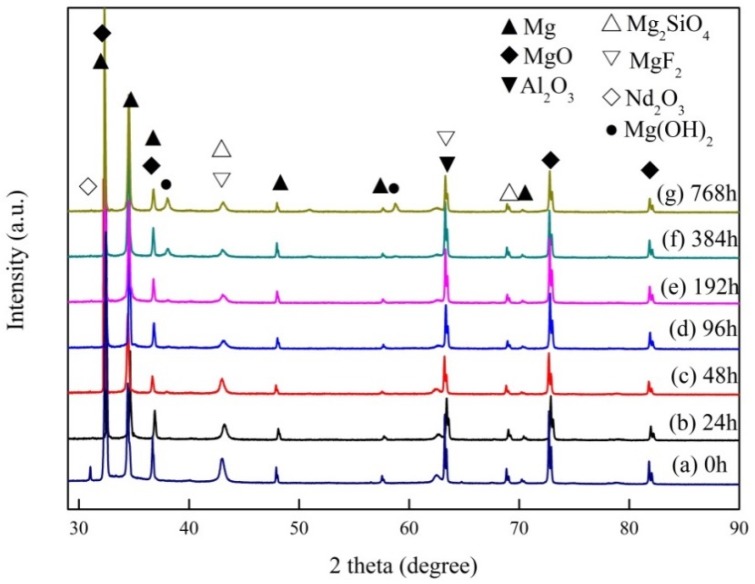
XRD patterns of (350–6) after immersion at different times.

Figure 9 shows the XRD patterns of (350–6) after immersion at different times. The main phases in the coatings changed a little after immersion. It is evident from Figure 9 that the coatings were mainly composed of MgO, Mg_2_SiO_4_, Al_2_O_3_, and MgF_2_. The peak of Nd_2_O_3_ was lost after immersion in the NaCl solution. After immersion for 384 h, the appearance of the peak of Mg(OH)_2_ in the coatings may demonstrate the corrosion product Mg(OH)_2_ produced by the reaction of magnesium with water. MgO reacts slowly with water, and the resulting Mg(OH)_2_ solids fall into the corrosion solution, so there were no Mg(OH)_2_ peaks detected prior to this. The peak areas of Mg_2_SiO_4_, Al_2_O_3_, and MgF_2_ exhibit a decrease, and the peak area of Mg increases with the increase in immersion time. This indicates that the film is becoming thinner and thinner, and the magnesium is being exposed step by step.

#### 3.3.2. Corrosion Resistance

**Figure 10:**
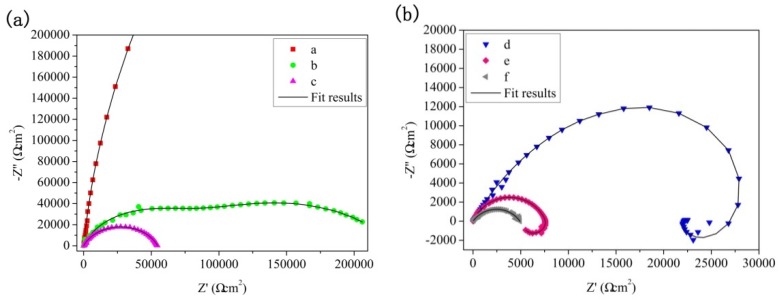

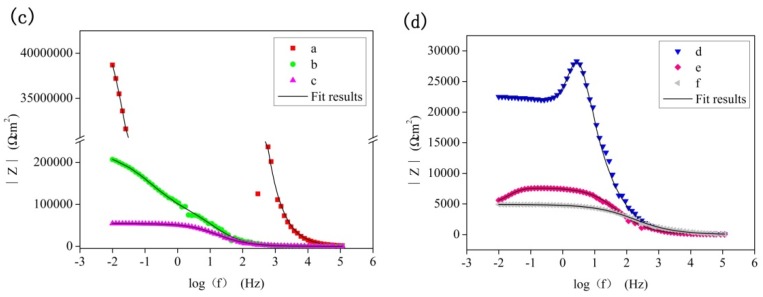
Electrochemical impedance spectroscopy (EIS) plots and fitting results of the MAO immersion coatings carried out in 3.5 wt % NaCl solution: (**a**,**b**) Nyquist plot, (**c**,**d**) Bode plot of Z vs. frequency. (**a**) 24 h, (**b**) 48 h, (**c**) 96 h, (**d**) 192 h, (**e**) 384 h, and (f) 768 h.

Figure 10 shows EIS plots and fitting results of the PEO immersion coatings carried out in 3.5 wt % NaCl solution. The corrosion rate was different owing to the greater radius of the capacitive loop [33,34]; the larger the radius of the capacitive loop, the lower the corrosion rate of the coating. It can be seen from Figure 10a that the coating exhibited high total impedance at the beginning of immersion, which indicated that the coating owned high corrosion resistance. Figure 10d is characterized by a capacitive loop in high and medium frequency ranges, and an inductive loop in the low frequency range, which was similar to the previous reports [35,36]. The appearance of capacitive loop is owing to the charge transfer process, whereas the inductive loop is related to the dissolution of Mg and is indicative of pitting corrosion of the substrate [37,38]. It can be seen from Figure 10a that during the initial immersion process, the capacitance loop shrinks rapidly, and the initial corrosion was mainly concentrated in the outer layer of the coating. With the immersion time increasing to 192 h, the low frequency inductive loop appeared. It was found in Figure 7e that obvious cracks appeared on the coating surface after immersion for 384 h, which indicated that the corrosion medium had reached the substrate through the crack.

**Figure 11:**
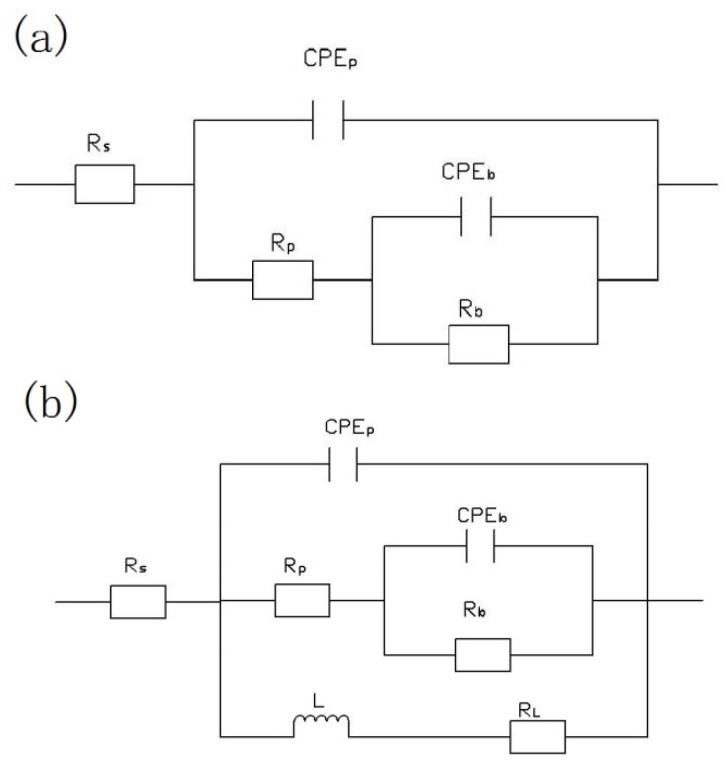
Equivalent circuits of the EIS plots at different immersion times: (**a**) 24 h, 48 h, 96 h; (**b**) 192 h, 384 h, 768 h.

The equivalent circuits of the EIS plots at different immersion times are shown in Figure 11. In the models, *R_p_*/*Q_p_* and *R_b_*/*Q_b_* are the resistance and constant phase elements of the outer layer and inner layer of the PEO coatings, respectively. *R* and the corresponding *Q* are parallel to each other. The constant phase element *Q*, a constant phase element (*CPE*), is used instead of a capacitor to compensate the non-homogeneity to obtain the best fit [28,39]. Further, n is a *CPE* exponent that provides a measure of the unevenness of the electrode surface; when n is equal to 1, the *CPE* behaves as an ideal capacitor. The corresponding equivalent circuit data are shown in Table 4. From Table 4, it can be concluded that in the early stage of soaking, the *R_p_* of the coating was higher, reaching 1.16 × 10^6^ Ωcm^2^, and the resistance of the barrier layer was 1.18 × 10^7^ Ωcm^2^. At this time, the corrosion resistance of the film mainly depends on the corrosion resistance of the dense layer [40,41]. This indicated that the inner dense layer of (350–6) coating plays an important role in the corrosion resistance. The values of *R_p_* and *R_b_* exhibit a decrease with the increase in immersion time, which indicated that the corrosion solution entered the coating through the pores, resulting in the destruction of the inner dense layer of the coating. The value of the inner layer resistance *R_p_* decreased continuously with the further increase in soaking time (192–768 h), which was caused by the corrosion medium entering into the inner layer of the coating and gradually eroding the dense layer. Figure 11b was used to fit the electrochemical corrosion of the coatings in this stage. At this stage, *R_L_* represents the charge transfer resistance of pitting corrosion with the inductance *L* [29].

#### 3.3.3. Degradation Mechanism Model

**Figure 12:**
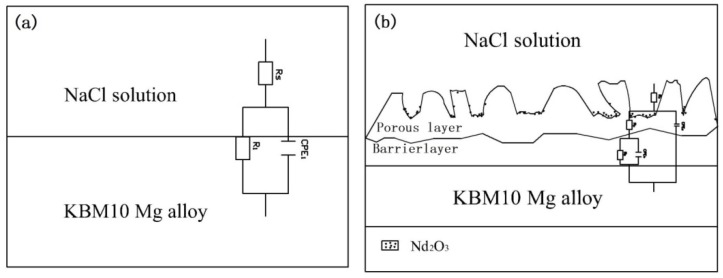
The model of KBM10 magnesium alloy substrate (**a**) and (350–6) PEO coating (**b**).

**Figure 13:**
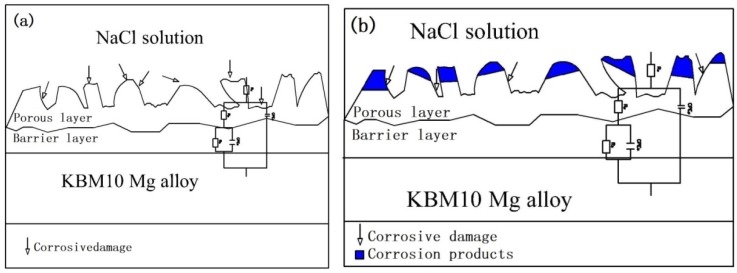

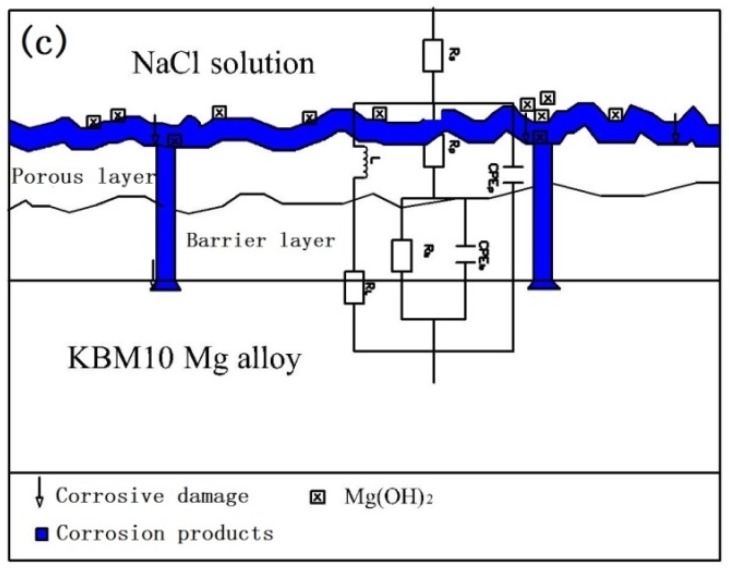
Degradation stage diagram of (350–6) in NaCl solution: (**a**) 24 h; (**b**) 48 h, 96 h; and (**c**) 192 h, 386 h, 768 h.

On the basis of the above experiments, the model of degradation was established such as Figure 12 and Figure 13. Figure 12a is the model of KBM10 magnesium alloy substrate. Figure 12b is the model of (350–6) PEO coating. After pretreatment, the thickness of the PEO coating increased, and the substrate was effectively isolated from the outside phase. (350–6) PEO coating included into porous layer and inner dense layer, and the dense layer played a major role in corrosion resistance. During the initial stage of soaking (Figure 13a), the film remains intact and largely undamaged. At the immersed middle stage (48 h, 96 h), the corrosion medium entered the interior of the coating through the pores on the coating. Nd_2_O_3_ on the surface of the film was corroded into the corrosion solution, and the MgO in the film layer participates in the reaction MgO + H_2_O = Mg(OH)_2_. In Figure 13b, the thickness of the coating decreased, and the corrosion medium gradually entered the coating through the pores. When the immersion time was increased to 192–768 h (Figure 13c), the corrosion of the film surface and its interior were corroded. The corrosion product Mg(OH)_2_ was deposited onto the surface of the coating gradually. The resistance of the porous layer increased (Table 4). At this stage, the corrosion medium was immersed on the substrate surface through the corrosion channel; the substrate will be destroyed, and the film will gradually relinquish the function of protecting the substrate.

## 4. Conclusions

The PEO coatings formed on magnesium alloys pretreated with Nd(NO_3_)_3_ solution at different concentrations were analyzed and tested. From the results and discussion above, some conclusions can be drawn as follows:

(1) The coating thickness exhibits an increase with increasing the Nd(NO_3_)_3_ solution concentration.
(2) The PEO coatings were mainly composed of MgO, Al_2_O_3_, Mg_2_SiO_4_, MgF_2_, and Nd_2_O_3_.
(3) The corrosion resistance of PEO coatings can be improved effectively pretreating the Nd(NO_3_)_3_ solution, and the best corrosion resistance was at the Nd(NO_3_)_3_ concentration of 0.06 mol/L.
(4) During the immersion tests, the main phase of the coatings, i.e., MgO, was transformed into Mg(OH)_2_ precipitated into solution.

## Figures and Tables

**Table 1:** Chemical composition of KBM10 magnesium alloy.

Element	Mg	Al	Zn	Mn	Li	Zr	Sb	Mo
wt %	Bal.	4–7	0.5–2.5	1–3	0.2–0.8	2–1.0	<1	<1

**Table 2:** The EDS result of MAO coatings.

Element	350–0		350–2		350–4		350–6		350–8		350–10	
	wt %	at%	wt %	at%	wt %	at%	wt %	at%	wt %	at%	wt %	at%
O	46.51	57.09	46.9	57.38	47.29	57.79	44.69	57.02	44.69	55.48	43.63	54.99
F	3.64	3.77	4.41	4.54	4.34	4.46	4.77	5.13	6.07	6.34	6.12	6.5
Mg	39.12	31.61	37.96	30.57	37.37	30.05	35.38	29.71	36.44	29.77	36.01	29.86
Al	1.33	0.97	1.26	0.91	1.15	0.83	1.3	0.98	1.42	1.05	1.09	0.81
Si	9.4	6.57	9.46	6.6	9.86	6.86	8.88	6.45	10.18	7.2	10.38	7.45
Nd	-	-	-	-	-	-	4.98	0.71	1.19	0.16	2.77	0.39

**Table 3:** Electrochemical parameters of bare alloy and MAO coatings specimens extracted from polarization test in 3.5 wt % NaCl.

Sample	*E_corr_*/(V vs. SCE)	*I_corr_*/(μA/cm^2^)	*β_a_*/(mV)	*β_c_*/(mV)	*R_p_*/(Ωcm2)
Bare alloy	−1.512	1.80 × 10^−4^	81.60	180.64	1.36 × 10^5^
(350–0)	−1.463	5.21 × 10^−5^	97.76	283.55	6.06 × 10^5^
(350–2)	−1.408	5.98 × 10^−6^	58.88	148.63	3.06 × 10^6^
(350–4)	−1.392	4.19 × 10^−6^	53.45	114.53	3.78 × 10^6^
(350–6)	−1.380	8.02 × 10^−7^	55.67	93.241	1.89 × 10^7^
(350–8)	−1.405	1.39 × 10^−6^	57.18	134.28	1.26 × 10^7^
(350–10)	−1.339	2.28 × 10^−6^	175.59	137.09	1.46 × 10^7^

**Table 4:** Equivalent circuit data of coatings immersed in 3.5 wt % NaCl solution.

Immersion time	*R_s_* (Ωcm^2^)	*Q_p_* (Ω^−1^ s^n^ cm^−2^)	n_1_	*R_p_* (Ωcm^2^)	*Q_b_* (Ω^−1^ s^n^ cm^−2^)	n_2_	*R_b_* (Ωcm^2^)	*L* (H)	*R_L_* (Ωcm^2^)
24 h	184.3	1.31 × 10^−9^	0.97	1.16 × 10^6^	8.43 × 10^−8^	0.52	1.88 × 10^7^	-	-
48 h	4.96	4.19 × 10^−7^	0.80	8.32 × 10^4^	2.56 × 10^−6^	1.00	3.48 × 10^4^	-	-
96 h	5.61	2.84 × 10^−7^	0.82	1.36 × 10^3^	6.12 × 10^−7^	0.70	5.42 × 10^4^	-	-
192 h	9.52	1.62 × 10^−6^	0.70	1.06 × 10^3^	3.41 × 10^−7^	0.78	3.65 × 10^4^	4.35 × 10^3^	5.79 × 10^4^
384 h	9.44	1.40 × 10^−6^	0.79	4.31 × 10^3^	1.98 × 10^−6^	1.00	3.06 × 10^3^	1.87 × 10^5^	1.44 × 10^4^
768 h	7.10	1.27 × 10^−7^	0.88	8.55 × 10^2^	7.64 × 10^−6^	0.58	3.97 × 10^3^	2.66 × 10^9^	2.44 × 10^9^
